# miRNA in blood-brain barrier repair: role of extracellular vesicles in stroke recovery

**DOI:** 10.3389/fncel.2025.1503193

**Published:** 2025-02-07

**Authors:** Vojtech Sprincl, Nataliya Romanyuk

**Affiliations:** ^1^Department of Neuroregeneration, Institute of Experimental Medicine of the Czech Academy of Sciences, Prague, Czechia; ^2^Department of Neuroscience, 2nd Medical Faculty, Charles University, Prague, Czechia

**Keywords:** acute ischemic stroke, mesenchymal stem cell, extracellular vesicle, exosome, miRNA, blood-brain barrier, tight junction, blood-brain barrier integrity

## Abstract

Ischemic stroke is a leading cause of mortality and long-term disability globally. One of its aspects is the breakdown of the blood-brain barrier (BBB). The disruption of BBB’s integrity during stroke exacerbates neurological damage and hampers therapeutic intervention. Recent advances in regenerative medicine suggest that mesenchymal stem cells (MSCs) derived extracellular vesicles (EVs) show promise for restoring BBB integrity. This review explores the potential of MSC-derived EVs in mediating neuroprotective and reparative effects on the BBB after ischemic stroke. We highlight the molecular cargo of MSC-derived EVs, including miRNAs, and their role in enhancing angiogenesis, promoting the BBB and neural repair, and mitigating apoptosis. Furthermore, we discuss the challenges associated with the clinical translation of MSC-derived EV therapies and the possibilities of further enhancing EVs’ innate protective qualities. Our findings underscore the need for further research to optimize the therapeutic potential of EVs and establish their efficacy and safety in clinical settings.

## 1 Introduction

Stroke is one of the leading causes of mortality and disability worldwide. In Latin America, one in ten patients dies within the first month after a stroke, and only six out of ten survive the first year (de Almeida Moraes et al., 2023). These statistics might differ in other countries due to more efficient healthcare systems. Globally, 11% of all deaths are caused by stroke, making it the second most common cause of death (Wang et al., 2016). The absolute number of stroke patients continues to rise due to the aging of the population. However, recent advancements in medication for high blood pressure have led to a decrease in stroke incidence in developed countries (WHO, n.d.).

Approximately 87% of strokes are classified as acute ischemic stroke (AIS) (Murphy and Werring, 2020). Current therapies heavily rely on tissue plasminogen activator (tPA) to dissolve blood clots, but tPA can damage the integrity of the blood-brain barrier (BBB), hampering stroke recovery (Qi et al., 2024; Donnan et al., 2008). Therefore, finding new approaches to treat AIS and restore BBB integrity is of utmost importance.

Mesenchymal stem cells (MSCs) have been studied for their potential for restoration of the integrity of the BBB following AIS (Qi et al., 2024; Tang et al., 2014). As of July 2023, 14 clinical trials focusing on MSCs’ clinical use after stroke were either ongoing or completed (Moñivas Gallego and Zurita Castillo, 2024). No clinical trials were registered considering MSC-derived extracellular vesicles (EVs) in stroke treatment on www.clinicaltrials.gov in August 2024. Given that MSCs likely cannot surpass a compromised BBB, it is hypothesized that their beneficial influence on BBB integrity is mediated through EVs (Bang and Kim, 2019). EVs are small lipidic particles produced by all cells. MSC-derived EVs (MSC-EVs) have become integral to current regenerative medicine. This review explores the potential of MSC-EVs in mediating neuroprotective and reparative effects, focusing on their miRNA cargo, and their role in enhancing angiogenesis and promoting restoration of the BBB integrity.

### 1.1 Ischemic stroke – pathophysiology

AIS is caused by blockage of blood vessels. Following the blockage, a so-called ischemic cascade starts (Xing et al., 2012). Glucose and oxygen deprivation leads to a switch from aerobic to anaerobic metabolism which causes an increased production of lactic acid that damages healthy neurons (Qin et al., 2022). Simultaneously, ineffective anaerobic metabolism does not produce enough ATP to support energy-demanding processes such as maintaining ion homeostasis. Cells become depolarized and cannot keep calcium out of the intracellular matrix, further depolarizing themselves. Glutamate transporters change their direction in reaction to depolarization and release glutamate into extracellular space (Heit et al., 2023). Extracellular glutamate travels to nearby NMDA (N-methyl-D-aspartate) receptors further spreading excitotoxic wave and calcium influx. Calcium activates mitochondria-induced cell death and causes overall damage to neuronal tissue (Rahi and Kaundal, 2024). This results in the transformation into necrotic tissue by the process called liquefactive necrosis (Chung et al., 2018). The afflicted area is called a cerebral infarct (Deb et al., 2010; Kristián and Siesjö, 1996).

**Figure 1:**
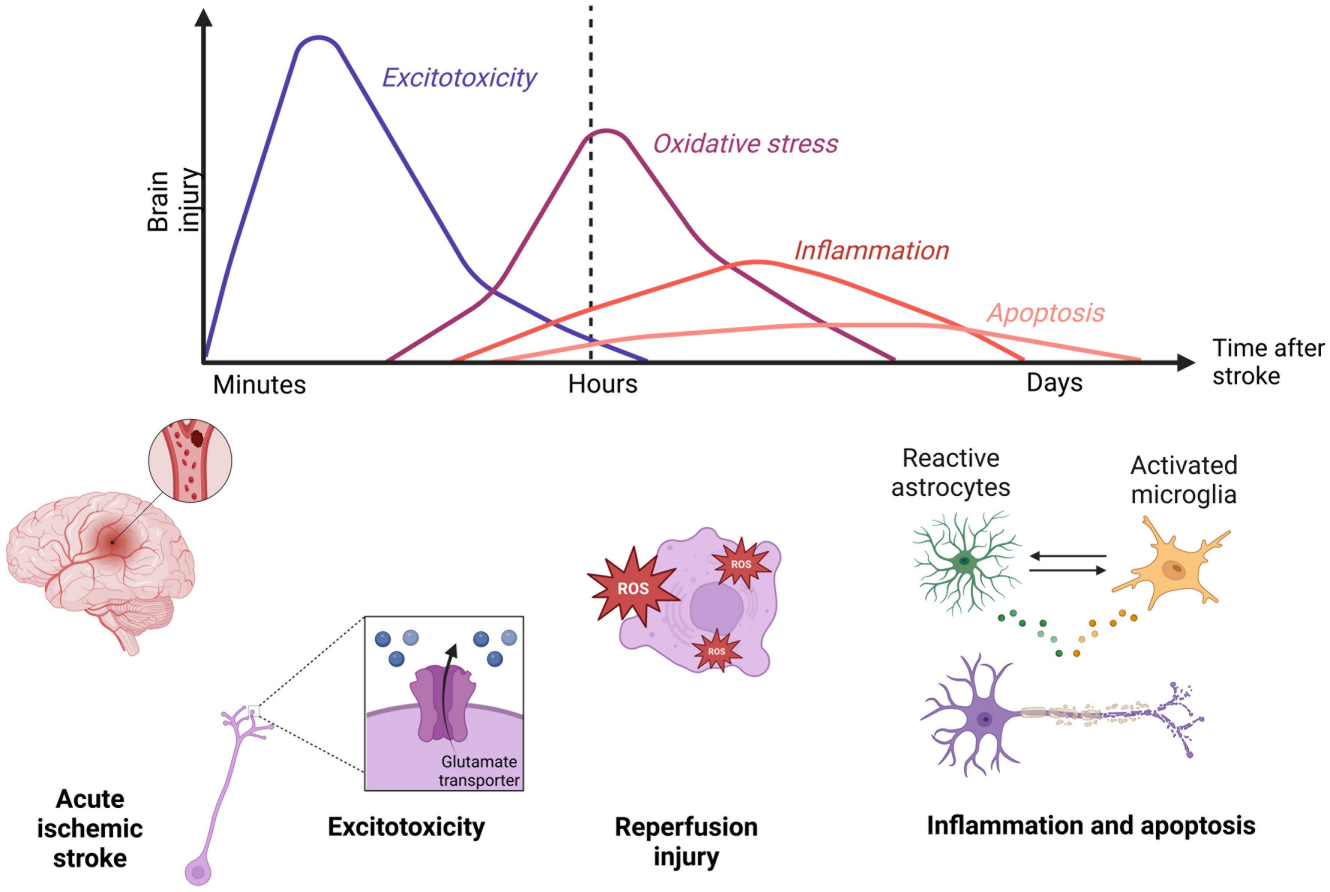
Timepoints in the ischemic stroke pathophysiology. Minutes following an acute ischemic stroke, an excitotoxic wave goes through ischemic tissue. After restoring the blood flow, a reperfusion injury characterized by oxidative stress and inflammation further damages the brain area. Created in Biorender.com.

Furthermore, as seen in Figure 1, a reperfusion injury occurs with the reintroduction of the blood flow. Reperfusion is characterized by increased oxidative stress and inflammation (Carden and Granger, 2000). Overall, necrotic neuronal tissue and reperfusion injury compromise the integrity of the BBB (Nian et al., 2020). Leaking BBB leads to edema, and increased permeability of neurotoxic substances, worsening the functional consequences of AIS (Chen S. et al., 2021).

The damage to the BBB mentioned above is a critical aspect of the pathophysiology of AIS. The BBB, a highly selective permeability barrier, protects the central nervous system (CNS) from potentially harmful substances circulating in the bloodstream. Understanding the structure and function of the BBB is essential for developing therapeutic strategies aimed at mitigating the adverse effects of ischemic stroke and enhancing recovery (Moñivas Gallego and Zurita Castillo, 2024; Bang and Kim, 2019).

### 1.2 Blood-brain barrier

The main cellular component of the BBB are endothelial cells (ECs). ECs form the inner wall of blood vessels and are connected by tight junctions (TJs). This is a system of proteins that close the space between individual cells, thus preventing the free passage of large molecules and ions. TJs are mainly formed by the proteins Claudin-5, Claudin-3, and Occludins (Profaci et al., 2020; Sweeney et al., 2019). Proper sealing of the BBB is crucial. Mice with complete depletion of Claudin-5 die within 10 days post-birth (Nitta et al., 2003). Depletion of transcription factor of TJs components resulted in microbleeding and dementia in adult mice (Weinl et al., 2015). TJs are linked to the cytoskeleton through scaffold proteins Zona occludens (ZO) -1, ZO-2, and ZO-3 (Obermeier et al., 2013; Profaci et al., 2020). Other important TJs proteins are vascular endothelial-cadherin and junctional adhesion molecules which further strengthen the integrity of the BBB (Li et al., 2018; Stamatovic et al., 2016). The BBB functions as a strict interface controlling the homeostasis of the brain via active transport. Passive diffusion is possible only for small, hydrophobic, and non-polar molecules. All other molecules have to go through specific transport. Transmembrane transporters are crucial for a healthy brain since they enable the delivery of nutrients. However, the same transporters can be troublesome for potential therapy (Wu et al., 2023). For example, aquaporins are hypothesized to cause edema after AIS (Chen S. et al., 2021). Additionally, ATP-binding cassettes are known to transport drugs out of the neuronal tissue. This is a major obstacle for various therapeutic molecules (Jiang et al., 2018). Both transporters are pivotal for a healthy brain but become an obstacle after AIS. ATP-binding cassettes transporters force us to search for a drug delivery way that can easily cross the BBB and will not be transported out from neuronal tissue (Wu et al., 2023).

**Figure 2:**
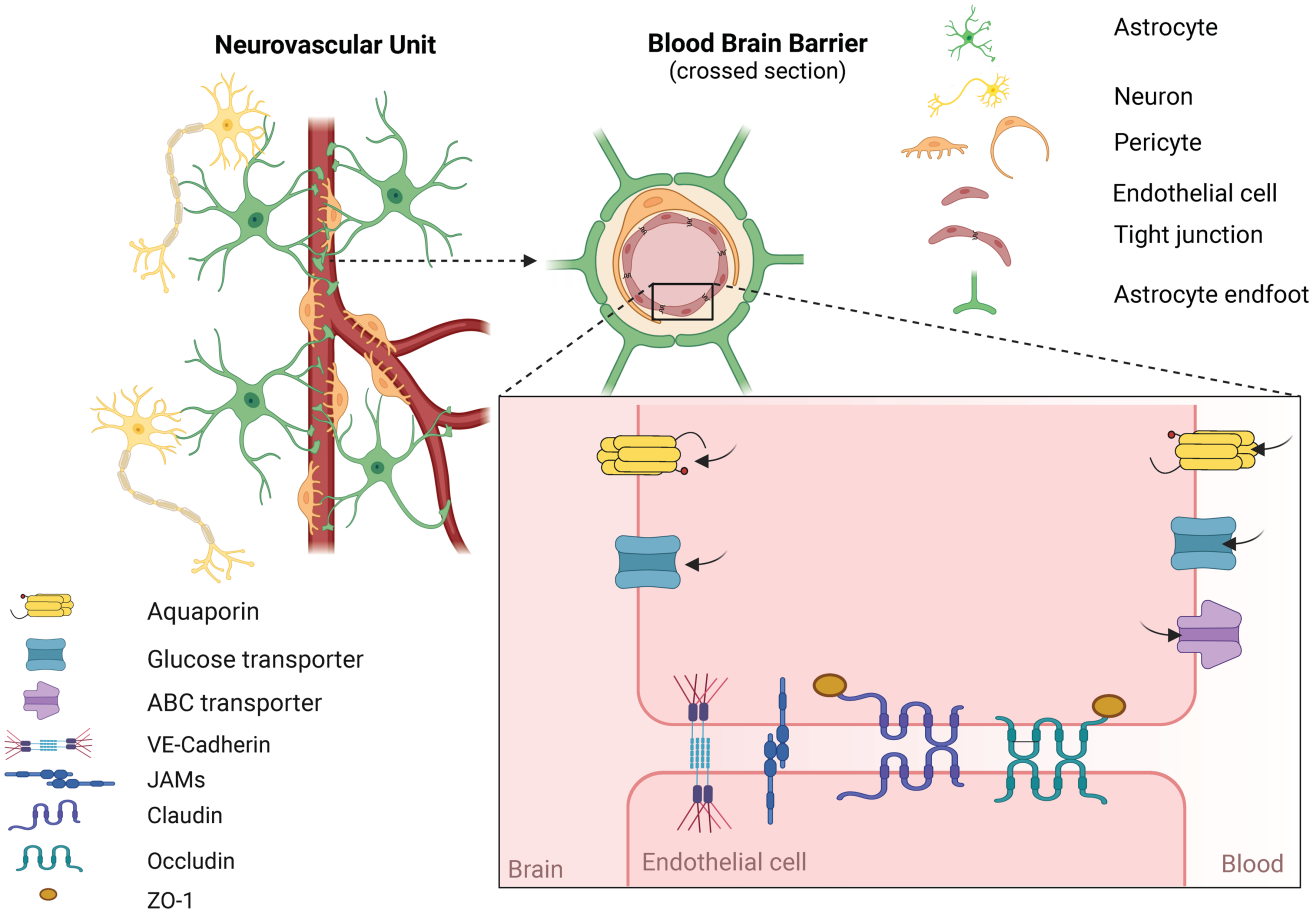
Structure of the neurovascular unit (NVU) and the blood-brain barrier (BBB). NVU consists of functionally interconnected cells. Namely endothelial cells, pericytes between which is a basal membrane. Further astrocytes, microglia, and neurons are part of the NVU. BBB is a narrower term and is typically formed by endothelial cells, pericytes, and astrocytes. Key components of the BBB are tight junction proteins (TJs) formed between touching endothelial cells. Proper sealing of the BBB via TJs allows for restricting the passage of harmful substances. Most molecules have to be transported via an active form of transport. Exogenous molecules for the NVU are tranposrted out of the brain via ABC transporters. Created in Biorender.com.

Equally important cell types forming the BBB are pericytes and astrocytes (ACs). ECs, pericytes, and ACs are in direct physical contact. On functional level, neurons and microglia also communicate with the BBB. Together they form a neurovascular unit (Wu et al., 2023). The structure of the BBB and neurovascular unit is shown in Figure 2. The function of pericytes is primarily the constriction of blood vessels, thus regulating blood pressure in capillaries. *In vitro* studies show that TJs protein expression is compromised in models lacking pericytes (Armulik et al., 2010; Bell et al., 2010; Winkler et al., 2011). Yet most *in vitro* studies do not use pericytes in their models (Qi et al., 2023; Eigenmann et al., 2013). ACs are supportive cells serving as intermediaries between neurons and the external environment of the CNS. The presence of ACs is crucial for the proper sealing of the BBB since they form astrocytic end-feet which further tightens the BBB (Wu et al., 2023). Mice with missing glial fibrillary acidic protein, one of the main components of the ACs cytoskeleton, have increased cerebral infarction area in a stroke model compared to wild-type mice which further underscores the protective role of ACs (Nawashiro et al., 2000). On the other hand, following AIS, ACs become activated (Liang et al., 2023). As such, they play a dual role. In one way, ACs are the source of neurotrophic factors such as angiopoietin 1 (Ang-1), Sonic Hedgehog, or Insulin-like growth factor 1 which have a positive influence on the integrity of the BBB. Activated ACs also reduce oxidative stress and brain edema, and overall help reduce the infarction area of AIS (Wu et al., 2023). But simultaneously, in reaction to the ischemic cascade, ACs produce metalloproteinases (MMPs). MMPs loosen the integrity of the BBB by degrading the extracellular matrix which is crucial for proper sealing of the BBB (Liang et al., 2023). ACs also form a glial scar which isolates injured tissue and prevents further spreading of the ischemic cascade, but afterward, the glial scar impedes regenerative processes (Shen et al., 2021). To simulate the AIS pathophysiology, most *in vitro* models of the BBB constrict of cocultivation of at least ACs with ECs. The presence of ACs is due to their complex role as important as the presence of ECs (Qi et al., 2023; Eigenmann et al., 2013).

The integrity of the BBB is crucial for maintaining the homeostasis of the CNS. Yet it poses a significant challenge for therapeutic interventions after AIS. The disruption of the BBB can exacerbate neuronal injury and impede recovery by allowing harmful substances into the brain parenchyma and at the same time pumps out the therapeutic ones (Wu et al., 2023). Other than during stroke, damage to the BBB occurs in various pathophysiologies such as multiple sclerosis, glioblastoma, or traumatic brain injury (Zierfuss et al., 2024; Kurz et al., 2022; Iqbal et al., 2024). Given the limitations of current therapeutic strategies that struggle to cross the BBB, innovative approaches are being explored. The general approach to repairing the BBB integrity would apply to multiple therapies (Rosenberg, 2012). One promising avenue is the use of stem cells and their products, which have shown potential in exerting neuroprotective and neurorestorative effects on the BBB. MSCs have previously been studied for their potential effects on restoring the integrity of the BBB following AIS (Qi et al., 2024; Tang et al., 2014).

## 2 The potential of mesenchymal stem cells in stroke treatment

MSCs are today’s most studied type of stem cells. The accessibility and lack of ethical concerns make MSCs an ideal candidate for allogenic or autogenic personal medicine. Currently, four types of MSCs are studied the most, bone marrow MSCs (BM MSCs), adipose tissue MSCs (AT MSCs), umbilical cord MSCs (UC MSCs), and Wharton’s jelly MSCs (WJ MSCs). Depending on the tissue of origin, it has been previously suggested that each subtype of MSCs could have specific properties and they should not be considered the same MSCs (Ding et al., 2011).

Intravenous administration of AT MSCs into rats injured by middle cerebral artery occlusion (MCAO), a standard murine *in vivo* model of AIS with subsequent reperfusion injury, led to a significantly better behavioral outcome (Mu et al., 2019). Additionally, increased BBB integrity was showcased by reduced Evan‘s blue signal in the injured area of the brain. Evan’s blue is a dye that is in physiological conditions unable to cross the BBB but can leak into neuronal tissue when the BBB is compromised (Saunders et al., 2015). Concurrently, a reduced cerebral infarct area after MSCs intravenous application was observed, suggesting an antiapoptotic effect of MSCs (Chen et al., 2015; Liu et al., 2022). MSCs have demonstrated promising therapeutic effects in enhancing BBB integrity and reducing damage to neuronal tissue after AIS (Do et al., 2021). In general, the regenerative effect of MSC is mainly attributed to their ability to create a supportive environment by releasing trophic factors (Kurozumi et al., 2005; Liu et al., 2018; Tanaka et al., 2018). Since MSCs most likely cannot cross even compromised BBB, it is generally hypothesized their beneficial influence on neurovascular unit and BBB integrity is mediated via EVs (Bang and Kim, 2019). As a result, studies of MSC-EVs have become a focal point for innovative and effective stroke therapies. Great obstacle to MSCs research is the variability between donors. For instance, primary cultures of AT MSCs from diverse donors vary considerably depending on the age, sex, and lifestyle (Fennema et al., 2009; Lee et al., 2015). Unfortunately, this variability most likely transfers into EVs research as well (Danielson et al., 2016).

### 2.1 Extracellular vesicles as miRNA carriers

EVs are produced by all living cells (Arif and Moulin, 2023). There are two main ways of EVs biogenesis. The more studied way is via multivesicular bodies (MVBs). MVBs are cellular compartments formed from endosomes via the inward budding of the endosome’s membrane. EVs are released into extracellular space after the MVBs fuse with the plasmatic membrane. These EVs are also known as exosomes (Han et al., 2022). Exosomes are typically 30–150 nm in diameter and have specific protein markers on their membrane. These markers are residues of biogenesis described above, namely, CD9, CD63, CD81, TSG101, and Alix (Théry et al., 2018). Another way of biogenesis is the outward budding of the plasmatic membrane. EVs produced this way are called microvesicles. Microvesicles have 50–1,000 nm in diameter, which means their size overlap with exosomes (Morales et al., 2016; Hessvik and Llorente, 2018; Kang et al., 2021). Unfortunately, there is no effective isolation method that could separate the overlapping fractions of exosomes and microvesicles. This also means that it’s difficult to determine whether exosomes or microvesicles are produced more. Therefore, the umbrella term „extracellular vesicles” has been agreed upon. Nevertheless, EVs are regularly defined as 30–200 nm in diameter with confirmed presence of said protein markers. This definition is generally recommended by Minimal information for studies of extracellular vesicles 2023 (Théry et al., 2018; Welsh et al., 2024).

EVs are known to contain proteins, mRNAs, and miRNAs as well as specific lipidic content of their membrane. EVs’ cargo partially reflects the characteristics of the cells of origin (Groot and Lee, 2020). In recent years, EVs have become the subject of numerous studies focusing on their inherent therapeutic and diagnostical potential (Kodam and Ullah, 2021). One of the most intriguing aspects of EVs is their ability to carry miRNAs. Together, miRNA and EVs are crucial for extracellular communication over long distances. The lipidic membrane of EVs protects miRNA content from endogenous RNases while the miRNA carries a piece of information (Boon and Vickers, 2013). miRNAs can modulate various cellular processes, including inflammation, apoptosis, and angiogenesis, which are crucial in AIS recovery (Xue et al., 2023; He et al., 2024; Guan et al., 2021). Measuring 20–24 nucleotides in length, they are a heterogeneous group of RNAs. miRNAs contain a seed sequence at their 5’ end, which enables them to bind to the 3′ untranslated region of the target mRNA (Lewis et al., 2003). One of the known mechanisms of miRNA regulation is the destabilization of the 3′ end of target mRNA, leading to a decrease in its translation (Eulalio et al., 2009). Generally, this regulation is more liake a fine-tuning, and the change in the expression of a single gene is altered only by a few percent (Selbach et al., 2008). However, it is important to mention that one mRNA usually contains multiple binding sequences and therefore can bind even dozens of miRNAs at once (Naeli et al., 2023). Nonetheless, even a relatively small change of expression of mRNA can lead to significant changes in a scale of whole cell. Similarly, miRNA-specific seed sequences can bind with multiple mRNAs, enabling one miRNA to regulate numerous translations simultaneously (Naeli et al., 2023). One complementary sequence can also be found on functionally related mRNAs that can be part of one signaling pathway. Alternatively, the protein products can interact, thus the effect of one miRNA can accumulate in this manner (Ichimura et al., 2011; Kehl et al., 2017).

A key characteristic of miRNAs is their strong tissue specificity, meaning that some effects could be diagonally different depending on the tissue (Hsieh et al., 2014). Lastly, because miRNAs come from a duplex of two pre-miRNAs, mature miRNAs have few isoforms. These are usually distinguished with the use of -3p or -5p. The effects of those isoforms could be vastly different (Bartel, 2018). It is therefore of utmost importance to be specific with the miRNA of interest and be guarded with the translation of knowledge from different tissues and pathophysiologies. In this review, we want to focus exclusively on miRNAs inside MSCs-derived EVs and their ability to protect the integrity of the BBB after AIS. Other than miRNAs, EVs can also be enriched with various allogeneic therapeutic molecules (Rädler et al., 2023; Gao et al., 2023). Some molecules are physiologically uptaken by the cells and become part of the produced EVs, other molecules have to be incorporated for example by electroporation (Rong et al., 2023). This is an advantageous way of delivery into the brain since EVs can easily cross the BBB (Rong et al., 2023). In the context of experimental models of stroke, EVs are typically either enriched with specific miRNA or the whole cargo is shifted using pre-treatment methods such as cocultivation with ischemic tissue extract. The ladder method is specific to MSC-EVs and this has been proven to improve their therapeutical potential significantly (Gregorius et al., 2021; Ye et al., 2022).

In this review, we accentuate the importance of miRNAs as a carrier of therapeutic potential of discussed MSC-EVs. It has been previously shown that miRNA carried by EVs regulate transcription factors activities and thus have enormous potential to mediate changes in target cells (Eirin et al., 2017). The curiosity and importance of miRNA in current research were highlighted this year with the Nobel’s prize awarded for the discovery of miRNA (Burki, 2024).

## 3 Mesenchymal stem cells derived extracellular vesicles in the models of stroke

In the following section, we would like to discuss the current knowledge of the therapeutic use of MSCs-EVs in the context of AIS with a particular focus on the repair of the integrity of the BBB. Here, we will focus on three main types of MSCs: BM MSCs, AT MSCs, and UC MSCs.

### 3.1 Bone marrow mesenchymal stem cells derived extracellular vesicles

Today’s most studied type of EVs are derived from BM MSCs (BM MSC-EVs). In general, BM MSC-EVs have neuroregenerative properties as shown by Doeppner et al. Treating MCAO-injured rats with BM MSC-EVs intravenously resulted in an increase in neuronal density as well as in an increased number of mature and immature neurons and ECs in the infarct area. More ECs suggest a possible angiogenic effect of BM MSC-EVs (Doeppner et al., 2015). A similar effect was later observed by Xu et al. with increased microvessel density as well as Ki67- and doublecortin-positive cells suggesting an ongoing angiogenesis and proliferation of neuroprecursors (Xu et al., 2020). Tian et al. studied how BM MSC-EVs affect the BBB integrity after MCAO injury in a mouse brain. After MCAO-induced injury, compromised BBB leaks fluorescein isothiocyanate (FITC) molecules into the brain. FITC is a fluorescent molecule that in physiological conditions, similarly to Evan’s blue, does not cross the BBB. Treatment with BM MSC-EVs via the tail vein led to reduced FITC signal in an MCAO-injured brain. This experiment showcases a great potential of BM MSC-EVs’ ability to restore the BBB integrity. The *in vitro* model of BBB further indicated angiogenic properties via increased trans-epithelial electrical resistance (TEER) values. TEER is an electrical resistance between two compartments, typically measured in a hanging insert with ECs and ACs cell cultures. The higher the value, the tighter the barrier. Increased TEER values were due to the miR-124 (Tian et al., 2022). miR-124 is one of the most expressed miRNAs in CNS (Han et al., 2020). Yang et al. transfected BM MSC-EVs with miR-124. Tail injection of miR-124 enriched EVs into MCAO-injured rats led to a decrease in the expression of SRY-box 2 (SOX2) and Nestin while inducing doublecortin expression (Yang et al., 2017). SOX2 is a marker of pluripotency and surprisingly, its depletion was shown to improve behavioral outcomes after stroke (Chen Z. et al., 2021). Changes in Nestin and doublecortin expression suggest a maturation of neuroprogenitors into neurons. Additionally, miR-124 has been shown to directly regulate peroxiredoxin 1 which in the context of stroke acts as a proinflammatory molecule by activating ACs (Tian et al., 2022). Furthermore, miR-124 inside BM MSC-EVs also directly regulates the mammalian target of rapamycin (mTOR) signaling pathway in ACs, resulting in upregulation in the glutamate transporter 1 (GLT-1) receptor (Huang et al., 2023). Overexpressed GLT-1 in striatum showed protective effects after MCAO injury in rats (Harvey et al., 2011). These studies suggest that BM MSC-EVs mediate a complex therapeutical effect on a neuronal tissue, especially on the BBB integrity.

Angiogenic properties of BM MSC-EVs have also been further studied by numerous authors which we will describe in the following text. The application of BM MSC-EVs on ECs led to an increase in the metabolic activity and cell viability (Hu H. et al., 2022; Bao et al., 2024). Boosted wound healing and migration were shown by scratch assay and transwell migration assay. Interestingly, Bao et al. used a scratch assay in oxygen glucose deprivation (OGD)/reperfusion condition and saw beneficial effects of used EVs. In a tube formation assay, wells treated with BM MSC-EVs had increased tube length and a significant increase in the number of branches (Hu H. et al., 2022; Bao et al., 2024). qRT-PCR analysis confirmed increased expression of let-7i-5p, miR-22-3p, miR-486 and miR-21-5p in ECs treated with BM MSC-EVs. miR-21-5p was upregulated the most. This indirectly suggests the presence of named miRNAs in BM MSC-EVs. The angiogenic effect was concurrently shown by increased expression of vascular endothelial growth factor (VEGF), VEGF receptor 2 (VEGFR2), Ang-1, and angiopoietin-1 receptor in the MCAO-injured mouse brain after BM MSC-EVs treatment (Hu H. et al., 2022; Bao et al., 2024). Upregulation of VEGF was also proven to be the effect of miR-210 which was artificially enriched in BM MSC-EVs by Zhang et al. Intravenous administration of miR-210 enriched BM MSC-EVs led to an increased survival rate of mice after MCAO injury. The same group also observed increased expression of CD34 which is a protein marker of bone marrow cells as well as ECs. Upregulation of CD34 thereby can mean elevated angiogenesis as well as just successful delivery of BM MSC-EVs. Interestingly, spoken EVs were additionally modified by linking c(RGDyK) protein onto their membranes. c(RGDyK) is a cyclo peptide that has a binding affinity to α_v_β_3_ integrins on cerebral endothelial cells. This modification led to a significantly increased concentration in brain tissue after intravenous administration compared to the naïve EVs (Zhang et al., 2019). Contrary to VEGF being considered as a proangiogenic marker, its effect after stroke has also been shown to be damaging the BBB integrity (Hu Y. et al., 2022). The group of Li et al. showcased reduced expression of VEGFR2 and VEGF-A after administration of BM MSC-EVs. Simultaneously, increased expression of TJ proteins occludin and ZO-1 was observed. Overall tightness of the BBB was measured via Evans blue and dextran leakiness *in vivo* and *in vitro*, respectively (Li et al., 2024; Li et al., 2023). Further research considering the role of VEGF in the BBB tightness after stroke is needed.

As already briefly mentioned above, BM MSC-EVs could serve as a cargo for therapeutic miRNAs. The beneficial effect of enriching BM MSC-EVs with certain miRNAs is an emerging new tool to boost the therapeutic effect of EVs even further. Yang et al. showed that the miRNA injected on its own did not reach neuronal cells. Meanwhile, electroporation of EVs with miR-124 led to the significantly more effective delivery of this miRNA into the injured region. This is mainly contributed to the EVs’ ability to cross the BBB while also protecting miRNA from degradation thanks to its lipidic membrane (Yang et al., 2017). Xu et al. observed that the majority of EVs at 3 days post-injection were in the liver and in the brain. The situation has changed 14 days post-injection when they moved to the gut (Xu et al., 2020).

Xin et al. enriched BM MSC-EVs with miR-17-92 by electroporation and injected intravenously these EVs into rats with MCAO-induced injury. Treated rats showed significant improvement in foot fault test and overall neurological severity score compared to untreated MCAO-injured rats 7 days post-injury. Delivery of miR-17-92 led to increased neurite branching and neural progenitor proliferation with reduced neural death. The beneficial effect of BM MSC-EVs delivery was also accompanied by a change in the expression of PTEN (Phosphatase and tensin homolog), pAkt (phosphorylated protein kinase B), mTOR and pGSK-3b (phosphorylated Glycogen synthase kinase-3 b) (Xin et al., 2017a). All proteins listed above, together with Wnt/b-catenin, are core components of signaling pathways, manipulation of which has been emerging in recent years as a promising way of restoring the integrity of the BBB (Mo et al., 2022). Lipofectamine transfection of EVs with miR-150-5p and later their stereotactic injection into MCAO-injured rat brains resulted in the regulation of toll-like receptor-5 and overall downregulation of the production of cytokines and apoptotic proteins (Li et al., 2022). Altogether results described above suggest that tailored BM MSC-EVs with specific miRNAs have greater therapeutic potential than naïve EVs.

Other than enriching EVs with exogenous miRNAs, MSCs positively react to hypoxic conditions which induce their proregenerative properties. This quality is also translated into the miRNA content of their EVs (Chang et al., 2023). Gregorius et al. showed that BM MSC-EVs released under hypoxic conditions contain more miR-126-3p, miR-140-5p, and let-7c-5p and less miR-186-5p, miR-30-3p and miR-409-3p compared to BM MSCs-EVs produced in normoxic conditions. Hypoxic conditions also led to alterations of protein content inside of EVs. In *in vitro* models of angiogenesis, hypoxic EVs proved to have prominently stronger angiogenic properties demonstrated in the conditions of the transwell migration assay and tube formation assay (Gregorius et al., 2021). Contrary to Hu et al. and Bao et al., who we mentioned above and who reported increased tube length, Gregorius et al. observed a reduced branch length which was compensated with increased tube density. This contradiction hints at a different effect of MSC-EVs produced in normoxic and hypoxic conditions (Gregorius et al., 2021; Hu H. et al., 2022; Bao et al., 2024). Other than cultivation in hypoxic conditions, 3D cultivation is also promising way how to increase BM MSC therapeutic potential. RT-qPCR analysis of MVBs showed different expressions of miRNAs in 2D and 3D cultures. 3D cultures produced MVBs, the yet unreleased EVs, with higher content of miR-10, miR-19a, miR-21, miR-22, miR-125b, miR-155, and miR-221 (Liu et al., 2023). We can hypothesize that MVBs would produce EVs with the same shifted content as studied MVBs. All the described studies using BM MSC-EVs were summarized in Supplementary Table S1.

In the preceding paragraph, we have already described the effect of miR-133b enriched EVs. Additionally, Xin et al. proved that BM MSC-EVs enriched with miR-133b significantly increased the release of astrocytic EVs *in vitro* and generally increased the number of EVs in the ischemic brain (Xin et al., 2017b). This secondary release of EVs can potentially further act therapeutically. Although, previously it has been shown that astrocytic EVs can act either positively or negatively, depending on the environment. For example, Wei et al. showed that ACs exposed to hypoxia produced EVs with higher content of miR-34c-5p, miR-141-3p, miR-200a-3p, and miR-140-3p. They further hypothesized that miR-200a-3p/miR-140-3p have a pro-inflammatory effect on ECs (Wei et al., 2023). Contrarily, Hou et al. showed that ACs under hypoxic conditions produce EVs containing miR-27a-3p which activate the Wnt/b-catenin signaling pathway through regulation of Rho GTPase activating protein. This was additionally proven to significantly induce the expression of Claudin-5, Occludin, and ZO-1 (Harati et al., 2022; Hou et al., 2024). Increased expression of TJs proteins indirectly hints toward tightening the BBB.

Together these studies open a new approach focusing on the secondary effects of the primary application of therapeutic BM MSC-EVs. This raises numerous questions. Is this secondary release of astrocytic EVs only the domain of miR-133b enriched EVs, or is it also a common quality of naive EVs? Additionally, how big of a protective impact is contributed to the primary introduction of therapeutic EVs and the secondary release of astrocytic EVs? Furthermore, is there a possibility that the secondary release of astrocytic EVs could potentially ameliorate the regenerative effect of the primary EVs considering that under certain conditions, ACs produce EVs with proinflammatory cargo? To make the topic more complicated, at early time points of AIS, proinflammatory EVs could have protective effects since an early stage of AIS is linked with immunosuppression (Santos Samary et al., 2016). As of today, these questions are being left unanswered, but they represent a potentially pivotal field in the regenerative application of EVs. ACs play a crucial role in the process of regeneration of neuronal tissue as well as in the protection of the BBB integrity after AIS.

### 3.2 Adipose tissue mesenchymal stem cells derived extracellular vesicles

AT MSC has been previously shown to have an angiogenic potential *in vitro* (Rautiainen et al., 2021). The same has also been proven with the AT-MSC-EVs. Increased tube length, total meshed area and number of branches was observed in a tube formation assay in wells treated with such EVs (Phelps et al., 2023). Moreover, AT-MSC-EVs isolated from cultured media in physioxic conditions (3% O_2_) demonstrated even stronger angiogenic effects. These physioxic EVs contained more VEGF-A than normoxic EVs, further complicating VEGF’s problematic dual effect. Additionally, EVs produced in a bioreactor had lower levels of Ang-2, fibroblast growth factor (FGF) -2, hepatocyte growth factor, IL-8, VEGF-A and VEGF-C, but they had more FGF-1. Such EVs had even better angiogenic properties (Liang et al., 2016; Phelps et al., 2023; Phelps et al., 2024). This should draw our attention to the conditions in which the EVs are produced, since potentially everything can strongly alter the properties of such EVs. It is then of the utmost importance to report every detail of cultured conditions openly (Welsh et al., 2024).

Yang et al. demonstrated that rat AT-MSC-EVs contained more miR-181b-5p after introducing MCAO brain extract to the culture medium (Yang et al., 2018). Later, Yang et al. observed an angiogenic effect of miR-181b-5p enriched AT-MSC-EVs using scratch assay and tube formation assay. They also validated that miR-181b-5p targets mRNA of transient receptor potential cation channel subfamily M member 7 (TRPM7), which is responsible for the excitotoxicity following AIS (Cepparulo et al., 2024; Yang et al., 2024) The angiogenic effect of miR-181b-5p was diminished when TRPM7 was overexpressed. Interestingly, the application of preconditioned AT MSC-EVs’ led to increased production of hypoxia-inducible factor 1 (HIF-1) and VEGF *in vitro*. HIF-1 and VEGF have been previously shown to damage the BBB integrity (Tsao et al., 2021; Yang et al., 2024). Even though the angiogenic potential of miR-181b-5p-enriched EVs on BBB has been proven, further research should be conducted to explore more broad mechanisms (Hu Y. et al., 2022; Shen et al., 2018; Yang et al., 2018).

Another group showed that tail vein injection of AT MSC into rats with MCAO injury improved BBB integrity via reduced Evans blue in the brain. Neuroprotection was measured by the reduction of the infarct area of the brain. Treated groups also had reduced expressions of bcl-2-like protein 4 (Bax), Bcl-2, and cleaved caspase-3 which further proves the antiapoptotic effects of AT MSC. ELISA assay showed downregulation of IL-1β, IL-6, and tumor necrosis factor α (TNF-α) compared to untreated control. Immunocytochemistry staining then showed upregulation of ZO-1, and claudin-5 in the MSC-treated group. Interestingly, the application of AT MSC led to decreased expression of miR-21-3p which has been shown to have proapoptotic and proinflammatory effects. Authors showcased that AT MSC-EVs’ downregulate miR-21-3p *in vitro* and thereby are likely to be the mediators of the described effects (Li et al., 2019). Finally, Rohden et al. applied human AT MSC-EVs’ to rats in the MCAO model and determined the effective dose to be 200 ug/kg. Subsequently, they measured the changes in BBB integrity via Evans blue assay and apoptosis by infarct area size. AT MSC-EVs’ had a positive effect on both. Treated rats had better behavioral outcomes in open-field tasks, novel object recognition tasks, and elevated maze tasks than untreated groups. Treated groups also improved angiogenesis and microvessel branching (Rohden et al., 2021).

AT MSC and EVs derived from them are studied the most for their angiogenic properties (Krawczenko and Klimczak, 2022). Unfortunately, only a few studies focus on the integrity of the BBB directly. This is a great scientific knowledge gap that should be the object of future research. AT MSCs are the most easily accessible of the four subtypes of MSC we mention in this review. They are also the best candidates for autogenic use in personalized medicine.

### 3.3 Umbilical cord mesenchymal stem cells derived extracellular vesicles

Last but not least, UC MSCs-EVs represent a lesser-studied type of EVs than the BM MSC-EVs or AT-MSC-EVs. However, that does not make them less promising. UC MSCs-EVs contain four times more miR-23a-3p than cell control of origin. miR-23a-3p regulates expression of Iba-1 (Allograft inflammatory factor 1), iNOS (cytokine-inducible nitric oxide synthases), TNF-α, and IL-6 together with an increase of Arg-1 expression in brain tissue of MCAO-injured rats. Administration of UC MSC-EVs has antiapoptotic and anti-inflammatory qualities shown by reduced infarct area size and reduced polarization of microglia into M2 phenotype. UC MSC-EVs’ effect has deteriorated with the use of miR-23a-3p inhibitor (Dong et al., 2022). The antiapoptotic effect was further enhanced when UC MSCs were cultivated with MCAO brain tissue extract. In agreement with previous research, neurological function was observed to improve more potently with MCAO brain extract-treated UC MSCs-EVs than with untreated ones. ECs culture damaged by the OGD, which is typically followed by reperfusion, were treated with UC MSC-EVs. Cell survival as well as Bcl-2 expression were increased meanwhile Bax and caspase-3 levels were reduced after the treatment. This further underlines the antiapoptotic boost of the hypoxia pretreatment method of cultivation (Ye et al., 2022). The antiapoptotic effect of UC MSCs-EVs was in a different study attributed to the presence of miR-24. miR-24 was simultaneously confirmed to target aquaporin 4 which is known to play a role in brain edema after stroke (Wang et al., 2021).

Additionally, Qiu et al. demonstrated that UC MSCs produce EVs that promote angiogenesis. Bioinformatic analysis predicted that the miRNA content of these EVs should alter Wnt and mTOR pathways. In a model of BBB, the administration of UC MSC-EVs led to a significant reduction of the damaging effect of tPA on the expression levels of Occludin and ZO-1. The protective role of UC MSCs-EVs on the BBB integrity was further shown by the decrease in Evans blue signal in MCAO-injured rats and reduced hemorrhage volume. In the *in vitro* model, Qiu and his team additionally demonstrated the protective role of UC MSC-EVs on BBB integrity by an increase in TEER values (Qiu et al., 2022).

Similarly to previous findings, Ye et al. showed that brain tissue extract preconditioning of UC MSCs leads to altered properties of the EVs they produce. Over two hundred miRNAs were differentially expressed between naive EVs compared to MCAO-tissue extract preconditioned EVs (UC MSC-I-EVs). We summarized this and all the described studies using AT and UC MSC-EVs in Supplementary Table S2. Furthermore, the difference in the efficacy of UC MSC-EVs and UC MSC-I-EVs was evident in a scratch assay. Cells treated with UC MSC-I-EVs demonstrated faster gap closure than naive UC MSC-EVs. More prominent protective effects were afterward shown when Ye et al. injected UC MSC-EVs into the tail vein of rats with ongoing MCAO injury. UC MSC-I-EVs had more potent neuroprotective effects when compared to unconditioned UC MSC-EVs. This was shown by the reduction of infarct area and number of TUNEL-positive cells. Both results imply a reduced apoptotic rate of neuronal cells. Interestingly, Ye et al. also studied the influence of healthy brain extracts on UC MSC-EVs characteristics. Preconditioning in this way also led to a more significant mitigation of neuronal apoptotic rate than with regular UC MSC-EVs, but not as prominent as with UC MSC-I-EVs. This trend was also evident in behavioral experiments. UC MSC-I-EVs treated MCAO rats improved their spatial and learning memory but also their speed in a maze (Ye et al., 2022).

## 4 Future perspective and challenges

Collectively, MSC-EVs are showing a promising future in regenerative medicine. All types of MSC-EVs exert to various degrees antiapoptotic, angiogenic, and anti-inflammatory properties, but there is no study comparing each type of MSC-EVs directly. Unfortunately, the studies of WJ MSCs-derived EVs that would prove their beneficial effect on the restoration of the BBB integrity are yet to be published. The research community faces the great task of deciphering molecular mechanisms of the positive effects of MSC-EVs. So far, in the context of AIS, there is only a limited number of studies focusing on BBB integrity. Additionally, future studies should elaborate on the options for preconditioning of MSCs. We have explored the possibilities of enriching MSC-EVs with certain miRNAs, but the same could be done with proteins or other therapeutic molecules. A protein modification of EVs can enhance the delivery to a specific site. Especially, AT MSC-EVs could potentially be used in personalized medicine. It has been shown multiple times that EVs do concentrate in the liver and gut, but so far, we do not know if this leads to any side effects.

Before any clinical use, there is an urgent need to optimize the MSC-EVs dosage as well as find a safe and effective way to cryo-store EVs. There are many options for this and the research community does not stand united in this manner. Some prefer to cryostorage EVs in −80 °C in PBS while others choose to leave EVs in the fridge. Experiments studying cryopreservants have also been conducted, but so far, they have not been introduced to good practice for the potential side effects. Nonetheless, EVs already have a great advantage over cell therapies, because EVs do not need DMSO for their cryopreservation (Bosch et al., 2016; Budgude et al., 2021; Charoenviriyakul et al., 2018; Qin et al., 2020). Another great obstacle before any clinical use is the scaling of the production. Even today, ultracentrifugation remains the golden standard for isolation of EVs. This is a lengthy and time-consuming protocol that is limited by the total media volume that could fit into the ultracentrifuge tubes. New methods, such as tangential centrifugation or size exclusion chromatography are slowly making their way into the field, but even they have their shortcomings (Théry et al., 2018). What we consider an obstacles the matter of dosing EVs. There is no consensus on which way it should be calculated. Some studies practice “total protein” or “protein concentration” dosing while others “total particle concentrations.” Furthermore, this does not unify either in *in vitro* or *in vivo* studies. While for *in vivo* experiments, the usual dosage ranges from 100–400 ug of EVs per rat, for the *in vitro* experiments, there is no consensus (Zhang et al., 2022; Waseem et al., 2023).
