# Supplementary material for: miRNA in blood-brain barrier repair: role of extracellular vesicles in stroke recovery
Source: Front Cell Neurosci. 2025 Feb 7;19:1503193. doi: 10.3389/fncel.2025.1503193 (PMC11842324; doi:10.3389/fncel.2025.1503193)
Supplement: Supplementary file 1 [file Data_Sheet_1.docx]

Supplementary Material

**Table 1: Overview of cargo and effects of bone marrow mesenchymal stem cells-derived extracellular vesicles in stroke**. *UC=ultracentrifugation, PEG=polyethylenglycol, ECs=endothelial cells, TEER=transepithelial electrical resistance, FITC=fluorescein isothiocyanate, GLT1=glutamate receptor, VEGF=vascular endothelial growth factor, VEGFR2=vascular endothelial growth factor receptor 2, ACs=astrocytes, MCAO=middle cerebral artery occlusion RhoA=Ras homolog family member A, OGD=oxygen glucose deprivation, PTEN=Phosphatase and tensin homolog, MMP=Matrix metalloproteinase.*

| **Vesicles source** | **Isolation method** | **miRNA cargo** | **Effect on stroke recovery** | **Reference** |
| --- | --- | --- | --- | --- |
| Human | Heparin, PEG, UC |  | *in vivo*:  ↑ neuronal density, ↑ ECs in the infarct area | Doeppner et al. 2015 |
| Mice | UC |  | *in vivo*:  ↑ microvessel density,  ↑ Ki67, ↑ Doublecortin | Xu et al. 2015 |
| Mice | UC | miR-124 | *in vitro*: ↑ TEER values  *in vivo*: ↓ FITC,  ↓ peroxiredoxin 1 | Tian et al. 2022 |
| Mice | UC | miR-124 | *in vivo*: ↑ Doublecortin,  ↓ SOX, ↓ Nestin | Yang et al. 2017 |
| Rat | ExoQuick | miR-124 | *in vitro*: ↑ GLT1 | Huang et al. 2023 |
| Rat | UC | Let-7i-5p, miR-22-3p, miR-486,  miR-21-5p | *in vitro*: ↑ metabolic activity of ECs, ↑ Tube length, ↑ wound healing,  ↑ migration assay  *in vivo*: ↑ VEGF, ↑ VEGFR2, ↑ Ang-1, ↑ Tie-2 | Hu et al. 2022 |
| Mice | UC | miR-210 enriched | *in vivo*: ↑ VEGF, ↑ CD34 | Zhang et al. 2019 |
| Rat | UC | miR-17-92 enriched | *in vivo*: ↑ neurite branching, ↑ proliferation of neural precursors,  ↑ foot-fault test,  ↓ neuronal death | Xin et al. 2017 |
| Rat | UC | miR-150-5p enriched | *in vivo*: ↓ cytokines,  ↓ apoptotic markers | Li et al. 2022 |
| Human | UC, PEG | Hypoxia-induced  ↑ miR-126-3p, ↑ miR-140-5p,  ↑ let-7c-5p, ↓ miR-186-5p,  ↓ miR-30-3p, ↓ miR-409-3p | *in vitro*: ↑ transwell migration assay,  ↑ tube formation assay | Gregorius et al. 2021 |
| Human | UC, PEG | 3D, MVB: ↑ miR-10, ↑ miR-19a,  ↑ miR-21, ↑miR-22, ↑ miR-125b,  ↑ miR-155, ↑ miR-221 |  | Liu et al. 2023 |
| Rat | UC | miR-133b enriched | *in vitro*: ↑ release of ACs-EVs | Xin et al. 2017 |
| Rat | UC | miR-486 | *in vitro:* ↑ cell viability of ECs; ↑ wound healing; ↑ branching length; ↓ PTEN | Bao et al. 2024 |
| Rat | Ultrafiltration, Exo-Prep kit |  | *in vitro:* ↑ cell viability of ECs ↓ permeability of FITC ↑ Claudin-5, ZO-1  *in vivo:* ↑ Claudin-5 and ZO-1 ↓ infarct volume | Li et al. 2023 |
| Rat | Ultrafiltration, Exo-Prep kit, UC |  | *in vitro:* ↓ MMP-2 and MMP-9  *in vivo:* ↓ Evan’s blue, ↑ behavioral outcome, ↓ VEGFR2, VEGF-A, CD147, and Caveolin-1 | Li et al. 2024 |

**Table 2: Overview of cargo and effect of adipose tissue and umbilical cord mesenchymal stem cells-derived extracellular vesicles in stroke.** *AT MSC=adipose tissue mesenchymal stem cells, PEG=polyethylene glycol, HIF-1=hypoxian-induced factor 1, VEGF=vascular endothelial growth factor, , FGF=fibroblast growth factor, UC=ultracentrifugation, UC MSC=umbilical cord mesenchymal stem cells, MCAO=middle cerebral artery occlusion, TEER=transendothelial electrical resistance, TGFB1=Transforming growth factor beta 1, Ang1=angiogenin 1.*

| **Vesicle source** | **Isolation method** | **miRNA cargo** | **Effect on stroke recovery** | **Reference** |
| --- | --- | --- | --- | --- |
| Rat AT MSC | PEG | miR-181b-5p enriched | *in vitro: ↑scratch assay,*  *↑ tube formation*  *in vivo: ↑ HIF-1, ↑ VEGF* | Yang et al. 2018 |
| AT MSC |  |  | *in vitro: ↓ miR-21-3p* | Li et al. 2019 |
| Human AT MSC | UC |  | *in vivo: ↓ Evan's blue,*  *↓ infarct area,*  *↑ behavioral recovery* | Rohden et al. 2021 |
| Human AT MSC  *physoxic conditions of cultivation* | UC |  | *in vitro: ↑* tube length, total meshed area and number of branches in tube formation assay, ↑ VEGF, TGFB1, Occludin | Phelps et al. 2023 |
| Human AT MSC  3D bioreactor | UC |  | *in vitro: ↑* content of FGF-1, FGF-2, VEGF-A and VEGF-C in 3D cultured EVs | Phelps et al. 2024 |
| Human AT MSC | UC | miR-125a | *in vitro: ↑* Ang1, *↑* tube length and number of branches in tube formation assay | Liang et al. 2016 |
| Human UC MSC | UC | miR-23a-3p | *in vivo: ↓ inflammatory cytokines, ↓ infarct area, ↓ M2 polarization of microglia* | Dong et al. 2022 |
| Human UC MSC | UC | MCAO induced content | *in vitro: ↑ scratch assay*  *in vivo: ↑ neurological functions, ↑ antiapoptotic markers, ↓apoptotic markers, ↓ infarct area,*  *↓ TUNEL positive cells* | Ye et al. 2022 |
| Human UC MSC | UC |  | *in vitro: ↑ TEER*  *in vivo:*  *↑ tight junction proteins,*  *↓ Evan's blue,*  *↓hemorrhage volume* | Qiu et al. 2022 |
